# A quantitative analysis of human rights-related attitude changes towards people with mental health conditions and psychosocial, intellectual, or cognitive disabilities following completion of the WHO QualityRights e-training in Ghana

**DOI:** 10.1186/s13033-023-00609-3

**Published:** 2023-12-05

**Authors:** Emma Poynton-Smith, Martin Orrell, Akwasi Osei, Sally-Ann Ohene, Joana Ansong, Leveana Gyimah, Caitlin McKenzie, Maria Francesca Moro, Nathalie Drew-Bold, Florence Baingana, Mauro Giovanni Carta, Priscilla Tawiah, Kwaku Brobbey, Michelle Funk

**Affiliations:** 1 Institute of Mental Health, Nottingham, UK; 2 Ghana Ministry of Health-Mental Health Authority, Accra, Ghana; 3 Charité University Medicine Berlin, Berlin, Germany; 4 WHO Country Office for Ghana, Accra, Ghana; 5 Nottinghamshire Healthcare NHS Foundation Trust, Nottingham, UK; 6 Columbia University Irving Medical Center, New York, USA; 7 World Health Organization, Geneva, Switzerland; 8 WHO Regional Office for Africa, Brazzaville, CG Congo; 9 Department of Medical Sciences and Public Health, University of Cagliari, Cagliari, Italy

**Keywords:** Human rights, People with disability, World Health Organization, Training, Ghana

## Abstract

**Background:**

Despite growing recognition of essential human rights, people with mental health conditions and psychosocial, intellectual, or cognitive disabilities’ rights are known to be frequently violated in mental healthcare worldwide, with common use of coercive practices and limited recognition of people’s right to exercise their legal capacity and make decisions for themselves on treatment and other issues affecting them. To tackle this issue, Ghana adopted the WHO QualityRights Initiative in 2019. This aims to introduce a right-based, person-centred recovery approach within the mental health care system, protecting and promoting the rights of people with mental health conditions, psychosocial, cognitive, and intellectual disabilities in the healthcare context and community.

**Methods:**

E-training (capacity-building) was provided in Ghana across a broad array of stakeholder groups including healthcare professionals, carers, and people with lived experience. The training covered legal capacity, coercion, community inclusion, recovery approach, service environment, and the negative attitudes commonly held by stakeholder groups; it was completed by 17,000 people in Ghana as of December 2021. We assessed the impact of the e-training on attitudes through comparing trainees’ pre- and post-questionnaire responses on 17 items, each measured on a 5-point Likert scale (strongly disagree to strongly agree), such that higher scores indicated negative attitudes towards persons with mental health conditions and psychosocial disabilities as rights holders. Analyses were conducted on two main groups: matched pairs (417 pairs of baseline and follow-up questionnaire responses matched to a high degree of certainty), and the unmatched group (4299 individual completed questionnaire responses).

**Results:**

We assessed the impact of the WHO QualityRights e-training on attitudes: training resulted in highly significant attitude changes towards alignment with human rights, with scores changing by approximately 40% between baseline and follow-up. In particular, attitude changes were seen in items representing treatment choice, legal capacity, and coercion. This change was not affected by age, gender, or background experience.

**Conclusions:**

The QualityRights e-training programme is effective in changing people’s (especially healthcare professionals’) attitudes towards people with mental health conditions and psychosocial, intellectual, or cognitive disabilities: this is a step towards mental healthcare being more with human rights-based worldwide.

## Background

People with mental health conditions and psychosocial, intellectual, or cognitive disabilities are especially vulnerable to violations of their human rights, contrary to the Universal Declaration of Human Rights (UDHR, 1948) [1] and United Nations Convention on the Rights of Persons with Disabilities (CRPD, 2006) [2–4].

Concerns about human rights violations in mental health services (which have been termed a “global crisis” [5]) exemplify the impact of poor funding [6, 7], longstanding stigma [8–16], the hegemonic approach of the medical model of disability, and the legacy of psychiatric care’s institutional history [17]. Coercive practices such as people being arbitrarily detained against their will, given medications without their consent, and even restrained (manually, chemically, or mechanically) or kept in isolation, remain embedded in mental healthcare across the world, even codified and justified in legislation [18–24]. There are also substantial reports in countries across the world of physical and sexual abuse as well as inability to access healthcare, vocational resources, and housing [25]. This is a systemic issue: the assumption that people with mental health conditions and psychosocial disabilities lack capacity to make decisions about their own lives and treatment is in line with both conventional mental health law and capacity-based law, in violation of the CRPD [26–28].

Low and middle income countries in Africa such as Ghana bear a disproportionate burden of mental health conditions; many share both traditional and Western approaches to mental health, each with distinct characteristics and their own associated stigma linked to social exclusion and discrimination [29, 30]. Such countries also often experience a huge gap in terms of financial and human resources in providing services: though provision has improved, as recently as 2012, people with mental health disorders in Ghana did not have access to basic mental health care and treatment [31]. Furthermore, assessments of Ghana’s mental health system in 2010 using the World Health Organization’s Assessment Instrument for Mental Health Systems (WHO-AIMS) identified high rates of coercive practices, leading to severe rights violations; similar findings were confirmed in a recent evaluation of psychiatric facilities across Ghana [30, 32, 33].

In an attempt to end these rights violations, Ghana in 2019 moved to adopt the WHO QualityRights Initiative (2012), a human rights model proposed by the CRPD, gradually shifting away from the medical and social models of disability. The WHO QualityRights Initiative aims to transform mental health system towards a person-centred rights-based approach and more generally to protect and promote the rights of people in services and the community. This is achieved through a comprehensive approach that includes capacity building, service transformation plans, policy, and legislative changes [34, 35].

As part of capacity building efforts, the QualityRights e-training program [28, 36] was created. The QualityRights e-training is a self-administered online course covering mental health human rights and how it applies in mental health, the recovery approach, respect for legal capacity, ending coercion, violence and abuse and community inclusion. It allows participants to start and finish the course at their own pace, usually taking between 8 and 15 h to complete, depending on the existing knowledge of the person completing the training.

Learning is undertaken across six relevant modules via various interactive formats including quizzes, videos, fact sheets, discussion forums, challenges and peer learning and coaching. In developing the QualityRights training programme the WHO worked closely with and sought extensive inputs from mental health professionals, people with lived experience, human rights experts, and mental health and organisations of persons with psychosocial disabilities [37].

The QualityRights e-training was rolled out on a national scale to key stakeholders including healthcare professionals, people with lived experience, carers, NGOs/OPDs, academics, students, and others. From February 2019 until December 2021, over seventeen thousand people in Ghana completed the e-training.

## Methods

### Aim

The aim of this study was to assess whether QualityRights e-training in Ghana impacted participants’ attitudes towards people with mental health conditions/psychosocial disabilities, intellectual or cognitive disabilities.

### Design and setting

Eleven key stakeholder groups within the Ghanaian mental health sector (both government and non-government organisations) oversaw the QualityRights initiative. These groups comprised the WHO Ghana, Mental Health Authority Ghana, Ghana Health Service, Mental Health Society of Ghana, MindFreedom Ghana, BasicNeeds Ghana, Inclusion Ghana, Ta-Excel Foundation, Passion for Total Care, Special Olympics, and the Christian Health Association of Ghana.

These eleven partners collaborated to promote the e-training across the country and encourage as many people as possible to complete the training. They disseminated the e-training widely, starting with recruiting their own staff and then spreading the e-training among their own target communities, partners, stakeholders and networks. Several dissemination strategies were used, including a launch event, social media promotion, forums, targeted promotional SMS messaging, prominent Ghanaian personalities creating videos as QualityRights champions, introduction in schools, and working with professional regulatory bodies to incorporate the e-training into Continuing Professional Development.

When signing up to the e-training, participants completed a form providing basic information including their name, contact information, country/state, affiliation, background experience and so on. They were also directed to complete a pre-course attitudinal baseline questionnaire before starting the e-training—a similar post-course questionnaire was sent to participants by email once they had completed the e-training. These questionnaires were developed by the World Health Organisation Policy, Law, and Human Rights Team through an informal consensus approach based on the main themes of the training, i.e., legal capacity, coercion, community inclusion, recovery approach, service environment, and the negative attitudes commonly held by stakeholder groups. The questions were reviewed and refined through several iterations of testing on colleagues for clarity, followed by a larger network of people to test basic psychometric properties.

The study is a pre-post evaluation of the improvement in attitudes, conducted both on the general sample of all those who completed the training and on a ‘matched’ sub-sample for which it was possible to identify the individual records and then evaluate the correspondence of the individual people. Training and evaluation were conducted over a time range with average.

### Participants

Participants in the e-training were key stakeholders within Ghana’s mental health system and the community, i.e., mental health and other care professionals, individuals with mental health conditions and psychosocial disabilities/their families, NGOs (non-governmental organizations), and other members or groups of the community more broadly: anyone with an interest was encouraged to sign up for the e-training.

The study participants consisted of people based in Ghana who undertook the e-training between February 2019 and November 2021 and filled the pre- and post-e-training questionnaire.

### Ethics

The WHO study gained ethical approval from the Independent Ethics Committee and the University Hospital of Cagliari, and approval by the Ghana Health Service Ethics Review Committee.

Participants were presented with a consent form on the e-training platform which informed participants about the aims of the training course, emphasising that participation was voluntary and they could stop or interrupt their training at any point (including withdrawing their data at any time). The data were kept confidential in accordance with the provisions that protect privacy in Ghana (Data Protection Act, 2012), and Articles Six and Nine of the EU Regulation.

### Measures/scoring

The pre- and post-course questionnaires were developed specifically by the WHO Policy, Law, and Human Rights Team for the QualityRights training: both contained the same 17 items (as listed in Table 1) relating to attitudes towards persons with mental health conditions and psychosocial disabilities. The questions were designed to incorporate themes around coercion, legal capacity, community inclusion, service environment, treatment choice, and hope. Demographic factors included age, gender, employment affiliation, and previous experience. It was devised using an informal expert consensus approach and showed good internal consistency, with a Cronbach’s alpha of 0.75.

**Table 1:** Sex and age descriptive statistics for both matched and unmatched groups

		Matched paired responses (N = 417)	Unmatched responses (N = 4299)
Sex (%)	Male	54.7%	52.1%
	Female	45.3%	47.9%
Age (years)	Mean (SD)	31.01 (6.50)	30.40 (6.89)

NB: Missing data excluded

Each item was measured on a 5-point Likert-scale (strongly disagree to strongly agree). All measured negative attitudes except three (items ‘g’, ‘m’, and ‘q’), which were therefore reverse-scored. For example, item ‘a’ stated “Nothing can be improved within mental health services without additional resources.” As such, higher scores indicated negative attitudes towards persons with mental health conditions and psychosocial disabilities as rights holders (not in compliance with human rights standards).

Total scores for the questionnaire were therefore calculated out of a total potential score of 68, where lower scores (down to a minimum of 0) indicate an approach in line with respecting human rights.

### Analysis

**Fig. 1:**
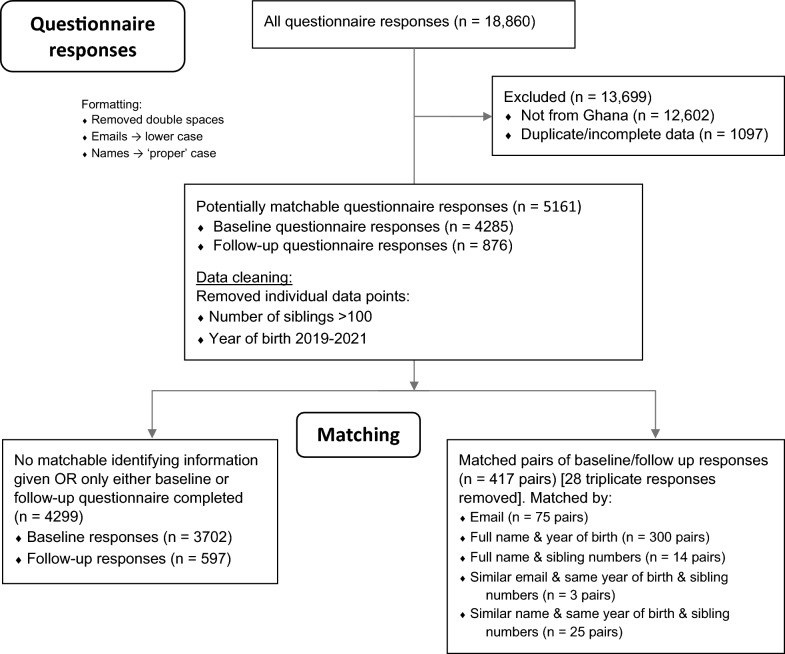
Flow chart showing process of identifying participants, data matching, and data cleaning

Data analyses (T-tests, Chi-squared, and multiple regression analyses) were conducted on IBM SPSS Statistics v27 (IBM Corp, 2020). Missing data were automatically excluded when the analyses were run. A significant amount of data clean-up was also necessary prior to analysis (reformatting to allow matching through text search, removing individual data errors such as ‘year of birth’ as the current year). The processes undertaken to narrow down the initial > 18,000 questionnaire responses are outlined in Fig. 1.

Our analyses were conducted on two main groups: matched pairs (417 pairs of baseline and follow-up questionnaire responses matched to a high degree of certainty using their email address/name/year of birth/number of siblings), and the unmatched group (4299 individual completed questionnaire responses, of which the majority were baseline). The matching process/grouping is also clarified in Fig. 1.

Of the original 5161 participants, 876 (17%) of the questionnaire responses represented follow-up data: most were baseline responses. This, along with identifying information not being a ‘required’ field (and the lack of a code number to pair questionnaire responses), limited the number of paired questionnaire responses which could reasonably be matched: over half of the follow-up questionnaires did not contain any useable identifying data (name/email).

## Results

### Demographics

Overall, 417 matched pairs were used for the pre- and post-e-training comparison. Their demographic information was broadly comparable to the 4299 unmatched Ghanaian questionnaire responses, as summarised in Table 1. For example, in terms of sex the matched pairs were 54.7% male and 45.3% female, while unmatched were 52.1% male and 47.9% female once missing responses where data were not missing. They were also similar in terms of age, with a mean age of 31.01 in the matched group compared to 30.40 in the unmatched group, as well as standard deviations being similar at 6.5 and 6.9, respectively.

The groups were also comparable in terms of their professional work: using only participants’ primary role response to avoid overlap, in both groups 70–80% of responses were from health or mental health practitioners. As can be seen in Table 2, there were some small differences in role distribution between the groups, especially a relative lack of responses from people with lived experience in the matched group, as well as fewer academics or administrators—this may suggest that such groups are less likely to share their personal information (to allow matching) or to complete the training and post-course questionnaire. It may be that mental health practitioners, who were somewhat over-represented in the matched group, were more likely to complete the training due to it being more directly relevant to their work. It is also worth noting that these analyses do not account for the overlap caused by dual category membership (e.g., mental health practitioners who also play an academic role).

**Table 2:** Primary background experience of questionnaire participants

	N (%)	
	Matched paired responses (N = 417)	Unmatched responses (N = 4299)
Mental health or related practitioner	207 (49.6%)	1544 (35.9%)
Health practitioner	124 (29.7%)	1446 (33.6%)
Academic	39 (9.4%)	578 (13.4%)
Administrator/manager	10 (2.4%)	176 (4.1%)
Human rights advocate	9 (2.2%)	127 (3%)
Person with lived experience/psychosocial, intellectual, or cognitive disability	6 (1.4%)	106 (2.5%)

NB: several categories too small for comparison to be made, including ‘Other/Missing’, ‘Student’, ‘Family member or care partner’, ‘Teacher’, ‘Policy Maker/Analyst’, ‘Social Worker’, ‘Person with other disabilities’, and ‘Lawyer’

### Attitude changes

**Fig. 2:**
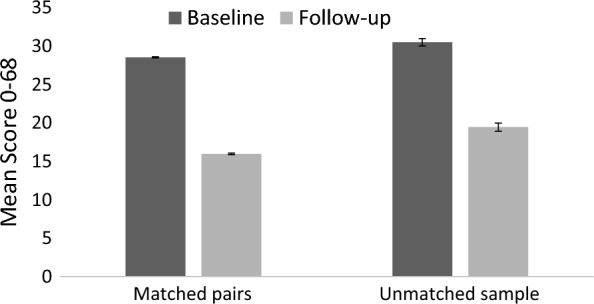
Attitudes before (baseline) and after (follow-up) completing WHO QualityRights e-training. Error bars indicate standard error of the mean (SEM), where a lower score indicates an approach in line with human rights

On average, comparing questionnaire responses available on all items, we found that participants’ attitudes significantly improved (i.e., lower scores, towards human rights-based approaches) after the WHO QualityRights e-training: this effect was maintained in the matched pairs group and the unmatched responses, as demonstrated in Table 3/Fig. 2.

**Table 3:** The significance of changes (chi-squared) in mean scores pre- and post-training

	Total score 0–68: Mean (SD)		Percentage improvement
	Baseline	Follow-up	
Matched paired responses (N = 417)	28.53 (9.90)	15.98 (10.79)	44.01^***^
Unmatched responses (N = 4299)	30.50 (9.29)	19.45 (11.85)	36.21^***^

^***^p < 0.0001

As shown in Table 4, paired samples T-tests demonstrated that differences in the matched pairs were highly statistically significant, with a large effect size: mean difference = 12.77, BCa 95% CI [11.74, 13.79], t(393) = 24.503, p < 0.0001, d = 1.23. An independent samples T-test also indicated a similarly significant difference in the unmatched group: mean difference = 11.04, BCa 95% CI [10.21, 11.88], t(4298) = 25.86, P < 0.0001, d = 1.14.

**Table 4:** Inferential statistics on overall attitude change following the e-training for matched and unmatched samples

	Mean difference (BCa)	p	d
Matched pairs (N = 394)	12.77 (11.74–13.79)	< 0.0001***	1.23
Unmatched (N = 4299)	11.04 (10.21–11.88)	< 0.0001***	1.14

^***^p < 0.0001

In terms of individual questions, a Chi-square test on all 5161 Ghanaian participants showed significant changes at the p < 0.0001 level for every question, as can be seen in Table 5, which also shows that the areas of most significant change in terms of individual questions were items j, g, o, and I (in italics). These items represented treatment choice, legal capacity, and coercion. All of these items showed an extremely significant reduction in mean scores, indicating a shift to attitudes in line with human rights, of over 45%. This effect was consistent when looking at the unmatched group as well.

**Table 5:** Proportion of scores, percentage change and significance (Chi-squared) of responses to every question between baseline and follow-up

		% of responses (417 matched pairs)					% Improvement in mean item attitude score	Pearson Chi-Square (*** = p < 0.0001)
		0 (SD)*	1 (D)	2 (Neutral)	3 (A)	4 (SA)		
a. Nothing can be improved within mental health services without additional resources	Baseline	12.7	42.5	3.8	26.2	14.7	21.05	79.71^***^
	Follow-up	31.7	39.8	5.0	15.8	7.7		
b. The service environment has little to do with people’s mental health and well-being	Baseline	33.2	42.3	4.1	16.3	4.1	14.50	53.35^***^
	Follow-up	47.2	34.1	3.6	12.2	2.9		
c. People with dementia should always live in group homes where staff can take care of them	Baseline	15.9	33.7	10.8	30.0	9.6	36.23	338.11^***^
	Follow-up	37.2	36.5	8.4	12.5	5.5		
d. People with psychosocial disabilities/mental health conditions should not be hired in work requiring direct contact with the public	Baseline	45.9	30.8	7.7	10.6	5.0	43.44	208.58^***^
	Follow-up	62.1	25.9	5.5	3.8	2.6		
e. Taking medication is the most important factor to help people with mental health conditions get better	Baseline	12.9	34.1	13.9	26.4	12.7	43.43	383.10^***^
	Follow-up	42.9	37.4	6.5	8.2	5.0		
f. You can only inspire hope once a person is no longer experiencing symptoms	Baseline	26.0	39.2	7.9	20.4	6.5	34.39	199.88^***^
	Follow-up	47.0	33.3	6.5	8.2	5.0		
g. People using mental health services should be empowered to make their own decisions about their treatment. [Reverse-scored]	Baseline	30.7	40.5	7.1	16.0	5.7	*52.90*	439.51^***^
	Follow-up	67.9	23.2	3.4	3.1	2.4		
h. Following advice of other people who have experienced mental health issues is too risky	Baseline	19.5	55.9	13.3	9.1	2.2	26.43	153.81^***^
	Follow-up	38.2	45.9	7.5	6.5	1.9		
i. The opinions of health practitioners about care and treatment should carry more weight than those of a person with an intellectual disability	Baseline	10.3	30.0	13.3	30.5	16.0	*47.69*	677.11^***^
	Follow-up	41.3	32.9	8.2	13.5	4.1		
j. It is acceptable to pressure people using mental health services to take treatment that they don’t want	Baseline	36.4	41.8	7.6	10.1	4.2	*57.66*	388.47^***^
	Follow-up	70.0	22.7	3.9	2.4	1.0		
k. Persons with mental health conditions should not be given important responsibilities	Baseline	34.0	45.3	7.4	8.9	4.4	44.00	229.45^***^
	Follow-up	58.0	32.1	4.6	3.9	1.4		
l. When people experience a crisis, health practitioners or families should make decisions based on their ideas about what is best for them	Baseline	9.7	19.5	7.0	45.9	18.0	42.32	576.12^***^
	Follow-up	37.6	26.1	6.3	23.4	6.6		
m. People with intellectual disabilities have the right to make their own decisions, even if I don’t agree with them. [Reverse-scored]	Baseline	18.7	48.1	13.2	16.7	3.2	40.13	422.26^***^
	Follow-up	50.2	38.3	3.4	5.9	2.2		
n. Controlling people using mental health services is necessary to maintain order	Baseline	11.7	25.9	17.0	33.7	11.7	40.57	455.79^***^
	Follow-up	39.8	32.2	7.1	16.3	4.6		
o. The use of seclusion and restraint is needed if people using mental health services become threatening	Baseline	8.2	12.5	8.5	45.1	25.7	*49.81*	860.60^***^
	Follow-up	39.5	27.6	9.0	16.8	7.1		
p. People at risk of harming themselves or others should be isolated in a locked room	Baseline	18.7	29.9	8.5	27.4	15.5	41.62	290.92^***^
	Follow-up	40.5	34.1	6.6	14.4	4.4		
q. Involuntary admission does more harm than good [reverse-scored]	Baseline	7.5	20.2	16.7	40.4	15.2	32.09	439.55^***^
	Follow-up	35.6	31.5	8.0	16.6	8.3		

*Where 0 indicates ‘strongly disagree’ for all items except reverse-scored, where it indicates ‘strongly agree’. A lower score always indicates an attitude in line with human rights

^***^p < 0.0001

In terms of predicting which participants’ attitudes underwent the most significant change, we did a forced entry multiple regression (shown in Table 6) on the paired samples based on change in total score (0–68). This did not show any significant effect of age, gender, or background experience (N = 387, df [3, 383], p > 0.05).

**Table 6:** Multiple regression analysis investigating impact of participant demographics on attitude change

Model	R^2^	Std. error of the estimate	F change	Sig. F change
Age, gender and experience	0.009	10.29	1.152	0.328 (p > 0.05)

## Discussion

This novel study demonstrated the positive impact of the WHO QualityRights e-training programme in Ghana for addressing stigmatising and discriminatory attitudes towards people with mental health conditions and psychosocial, intellectual, or cognitive disabilities and for aligning attitudes with a human rights-based approach. In particular, participants (who comprised mental health/healthcare professionals, academics, administrative staff, and people with lived experience) completing the e-training programme showed highly significant attitude changes aligned with human rights across all items, with scores changing by approximately 40% between baseline and follow-up. This change was unaffected by sex, age, or background experience.

This result is especially impactful given the nature of the e-training provided: uptake of the e-training highlights that online training is accessible to a large number of people even in lower income countries, and this accessibility suggests that the e-training could easily be introduced into the curricular training of health professions’ degree programmes, or staff training programmes.

Generally, the impact of training programmes can be measured in terms of effect on knowledge, attitudes, and practice: they do not always co-occur. In this case, we know that knowledge has improved, as demonstrated by progression through the e-training course and successful completion of the continuous assessment elements of each module, and the current study provides strong support for an impact on attitudes. It is often assumed that attitudes are largely predictive of behaviour (per the Theory of Reasoned Action), but evidence on the attitude-behaviour relationship tends to show low correlations at best [38]. Therefore, the question remains in this case as to whether attitude changes are predictive of practice change.

The idea that people who are familiar with (i.e., knowledgeable about) human rights will believe in and commit to upholding others’ rights [39] (i.e., change their attitudes and practice in line with this knowledge) has face validity, and there is evidence that knowledge of human rights makes support for people using services more effective, as well as that specific human rights training is beneficial for people with lived experience, staff, and organisations [40]. A meta-analysis of attitudes’ prediction of behaviour concluded that attitudes were more predictive if they were strong beliefs, stable over time, easy to recall (accessible), and relevant to their behaviour (i.e., they had direct experience of related situations) [41].

A qualitative synthesis of ten human-rights based approaches (HRBAs) in mental healthcare settings (through various training interventions/clinical decision-making guides) suggested that HRBAs can lead to behaviour (practice) change, as they showed clinical improvements at relatively low costs, contributing to positive therapeutic outcomes such as treatment satisfaction for people using services, their increased involvement, reduced use of seclusion, improved hygiene, improved health check access, reduced falls incidence, reduced need for anti-psychotic medications, and reduction in severity of challenging behaviour [25].

As such, we could expect human rights training to alter attitudes, and for this to be predictive of behaviour, especially for people with personal experience of human rights violations and for those working in settings where human rights violations occur. In a qualitative investigation of particular relevance to the current study, Human Rights Watch conducted seven interviews with Ghanaian mental health professionals, most of whom had completed the QualityRights e-training, and found a marked shift towards human rights-based approaches in both attitudes and practice in Ghana [42]. Consequently, there is substantial evidence that human rights-based training can impact both attitudes and behaviour, causing changes in practice and therapeutic outcomes.

The results of the current study in terms of capacity building are particularly important in the context of changing the paradigm of mental health care: the same Human Rights Watch article reported that coercive practices are in the process of reducing, with more awareness that mental health is not simply about taking medications or keeping patients secluded to reduce risk of harm—interview responses showed a shift to a person-centred, human rights-based approach to care [42]. The WHO QualityRights training therefore has the potential to change coercive practices and promote a new, human rights-focused paradigm of care.

Ghana represents an excellent example when considering the wider implications of this work, as the problems their mental healthcare system faces are comparable (albeit with their own specificities) to other countries across the world, e.g., underfunding, societal stigma, discrimination, and human rights violations, including the use of coercive practices and denial of legal capacity amongst others. The WHO QualityRights Initiative e-training was made available worldwide without need for an access code in April 2022, and as such many other countries may follow in Ghana’s footsteps, with many hundreds of thousands of healthcare professionals (and anyone with an interest) able to benefit from the e-training.

Nevertheless, there are some limitations. Firstly, our sample was limited by our sampling procedure: while the e-training is now open to anyone who can access it online, at the time of data collection it was limited to people who had been given an access code through national dissemination across Ghana. The sample is therefore biased, representing participants with connections to key stakeholder groups in Ghana—this could go some way towards explaining why the vast majority of our participants (80%) were healthcare professionals. This may limit the generalisability of our findings across all groups.

For example, the paucity of participants who identified as having lived experience (even more pronounced in the matched sample) unfortunately limits the breadth of our analysis in applicability to attitudes in people with mental health conditions and psychosocial, intellectual, or cognitive disabilities themselves. This may represent either stigma in sharing identifying information (hence reduced numbers in the matched groups, if they were less willing to share identifiable details) or may indicate that people with lived experience are less likely to complete the e-training; future research exploring the accessibility of the e-training for people with lived experience would be useful. Conversely, the increased proportion of mental health practitioners in the matched sample may suggest that they were more likely to complete the training (and give identifiable personal details) because of its relevance to their work or because it was required by their employer/paid. This is a limitation which could be easily addressed in future research, especially now that the e-training is available to the general public: the only limitation will be information dissemination.

Furthermore, there were limits in our data collection methods resulting in a relative lack of follow-up questionnaire responses, as well missing identifying information allowing us to match up baseline and follow-up responses. This limited the potential sample size and resulted in separate analyses being conducted for both the matched and unmatched responses, in order to make best use of the data available. Furthermore, as the questionnaires were not mandatory at either baseline or follow-up, there are many potential participants for whom outcomes from the e-training have not been measured—this is a missed opportunity in terms of data collection, and could have resulted in reduced bias in our data collection, as well as a larger sample size.

Ideally, the questionnaires would have been embedded in the training as a mandatory component linked to the participant’s account, so that we could also access data on their interaction with resources and time taken to complete the training, as well as quiz scores, enabling more in-depth analysis: for example, the amount of time elapsed between completing the e-training and filling in the post-training questionnaire could have a significant impact on reported attitudes, but we are not able to link the questionnaire and e-training data directly. However, on the whole, we were still able to access a huge sample size, with over 5000 completed questionnaires from Ghana alone, and the sample size was more than sufficient given the size of the effect of the training.

Furthermore, the follow-up responses are likely to be biased as only those who have completed the e-training are given the link to the follow-up questionnaire, and so participants who found the training too difficult (or found it conflicted with their beliefs) are less likely to have completed the follow-up questionnaire, meaning the percentage change in attitudes may be somewhat inflated. There is also, as always, the impact of courtesy and social desirability biases, where participants are likely (especially after training) to give answers which reflect what they think the researchers would like to hear or what they feel they ought to say, rather than their true attitude or beliefs.

In future, it would be ideal if further research could take an integrated approach to assessing attitude change, including the pre- and post-training questionnaires as essential elements of the programme, linked directly to the user’s account to ensure both responses can be linked. It would also be extremely beneficial to have a direct comparison between outcomes on units where staff have and have not yet undergone the training, perhaps through an observational study on compliance with human rights-based approaches (e.g., frequency of coercive practices) or through service user satisfaction surveys.

## Conclusion

This novel study aimed to assess the effect of the WHO QualityRights e-training programme in Ghana on trainees’ attitudes towards people with mental health conditions and psychosocial, intellectual, or cognitive disabilities. The data suggest that, in our representative sample of trainees, participants in Ghana showed highly significant attitude changes to being more in line with human rights across the board, with scores changing by approximately 40% between baseline and follow-up (unaffected by demographic characteristics such as age, sex, and background experience).

This provides strong evidence that mental healthcare should aim to adopt human rights-based approaches, though further evidence to support the impact of changes in attitudes on changes in practice would add further support. It also validates the QualityRights e-training as an evidence-based programme for changing stigmatising and discriminatory attitudes towards people with mental health conditions, or people with psychosocial, cognitive, and intellectual disabilities and for improving alignment with international human rights standards.

## Data Availability

Anonymised versions of the datasets used and/or analysed during the current study are available from the corresponding author on reasonable request.

## Abbreviations

WHO: World Health Organization
UDHR: Universal Declaration of Human Rights
CRPD: Convention on the Rights of Persons with Disabilities
HRBAs: Human-rights based approaches
NGOs: Non-governmental organizations
WHO-AIMS: World Health Organization’s Assessment Instrument for Mental Health System
OPDs: Organization of persons with disabilities
